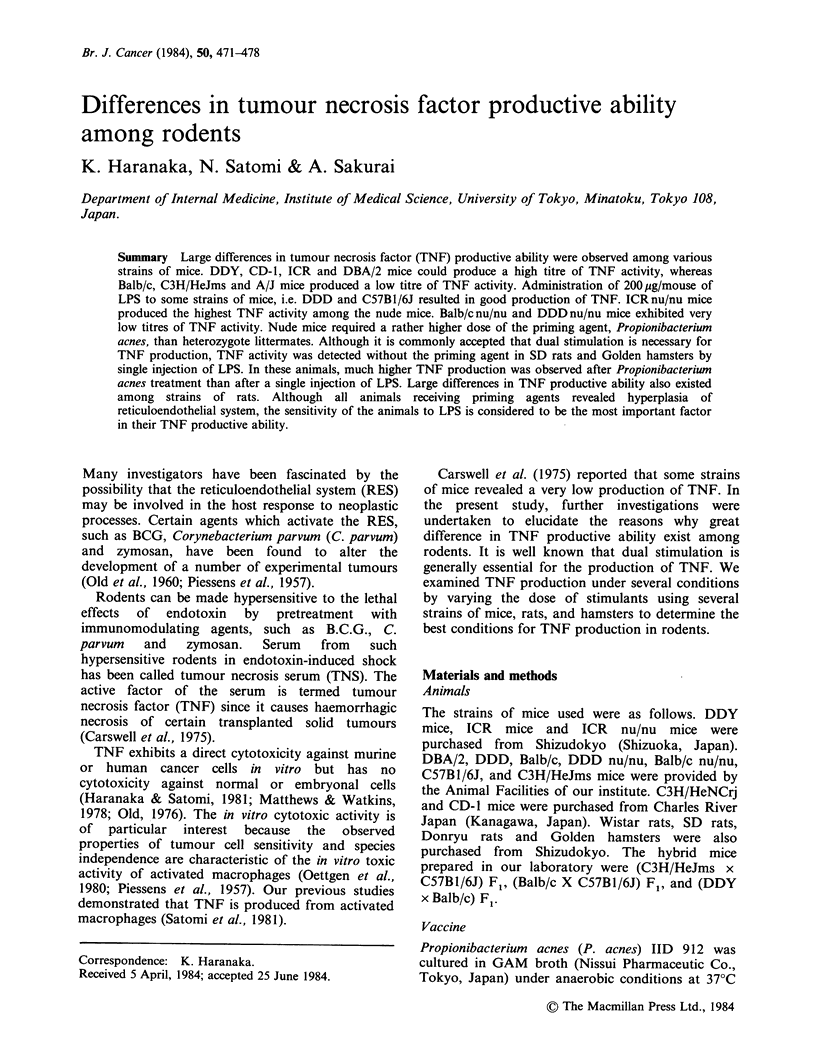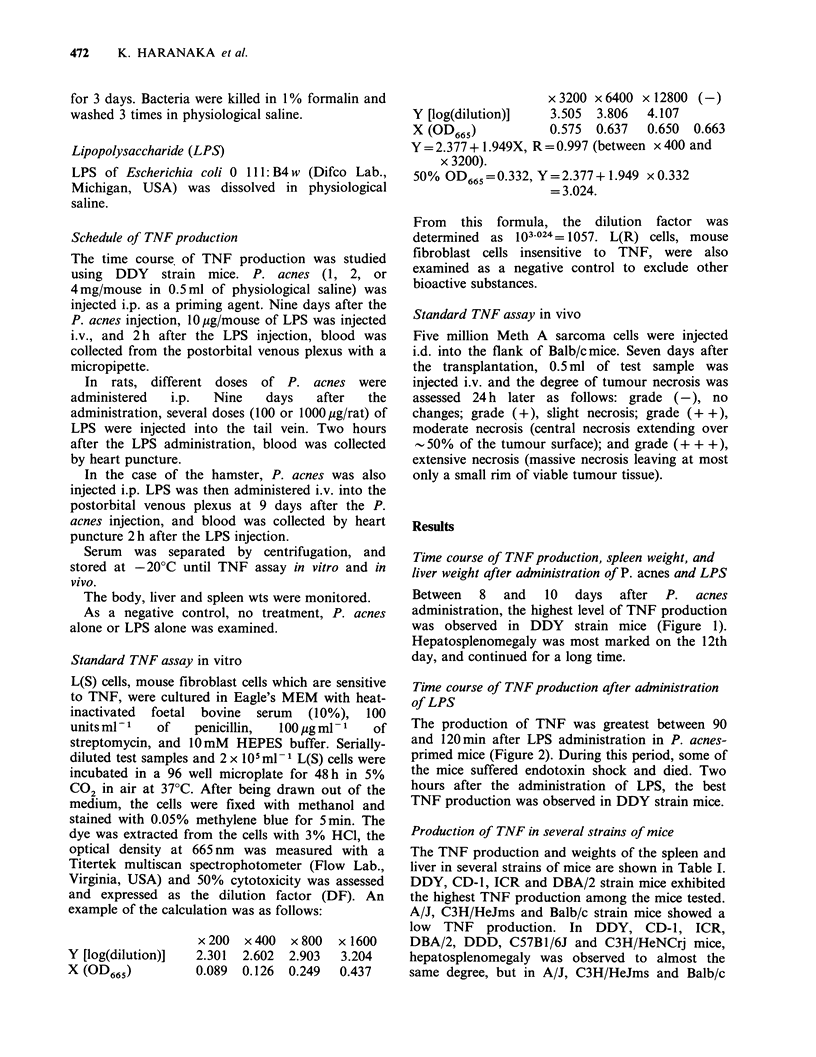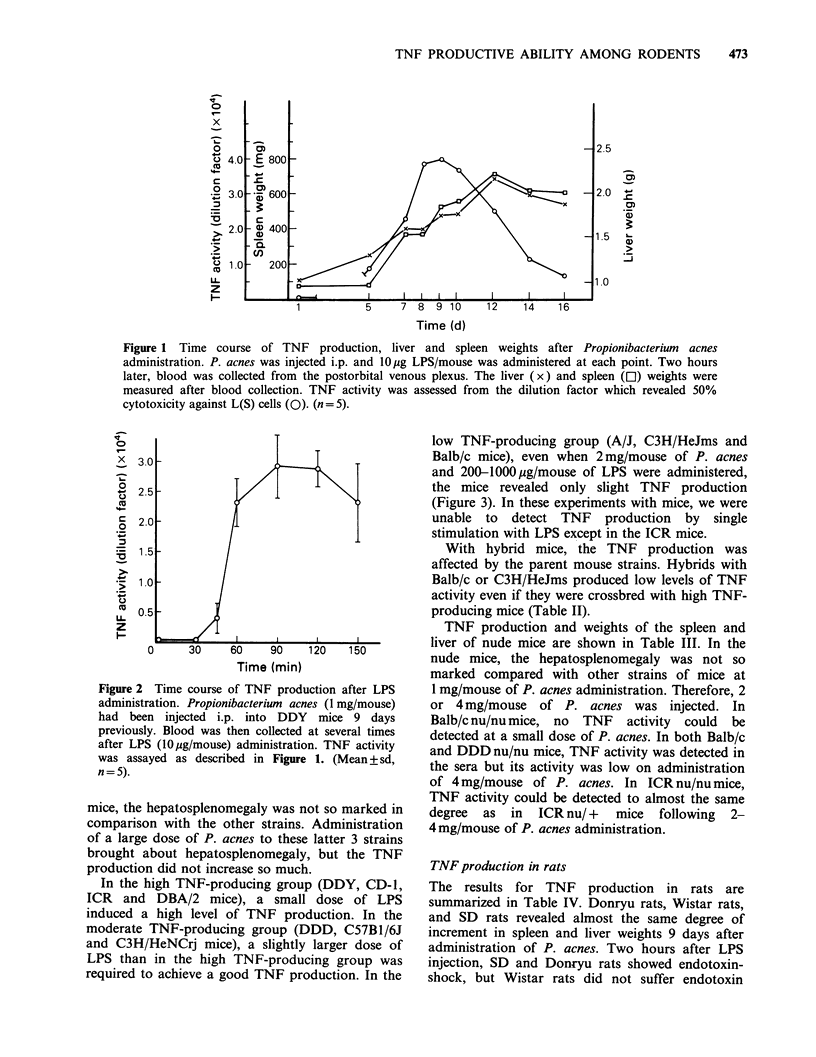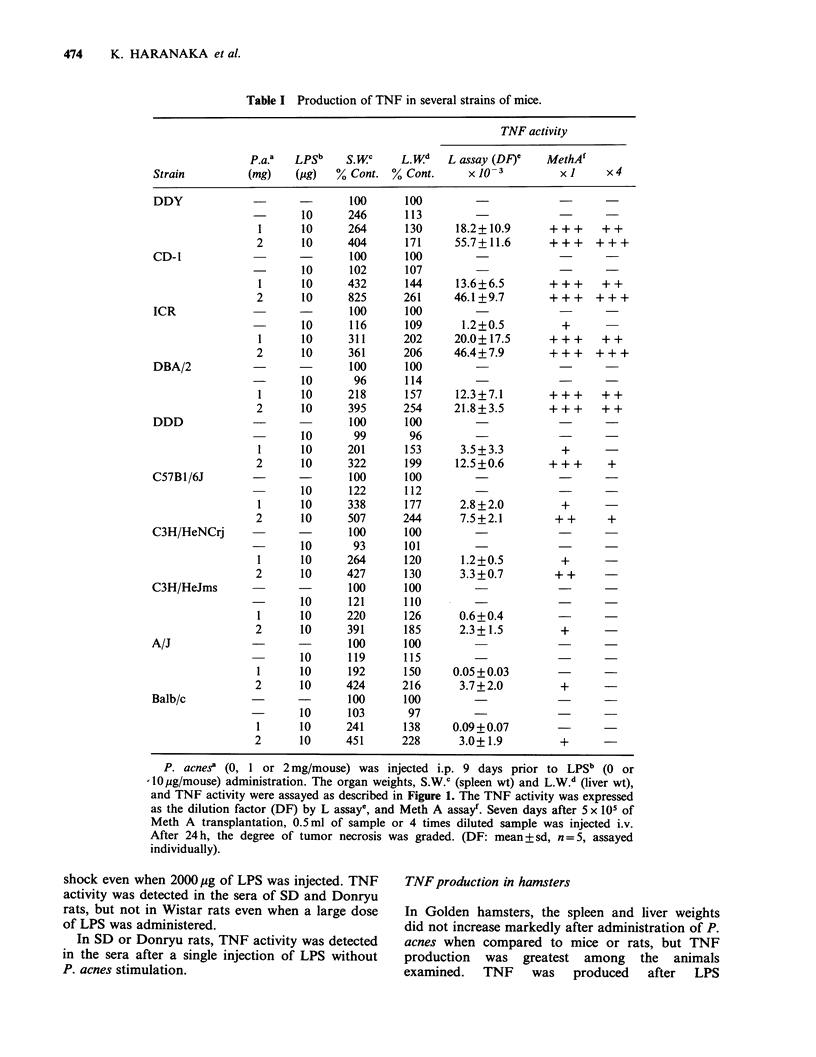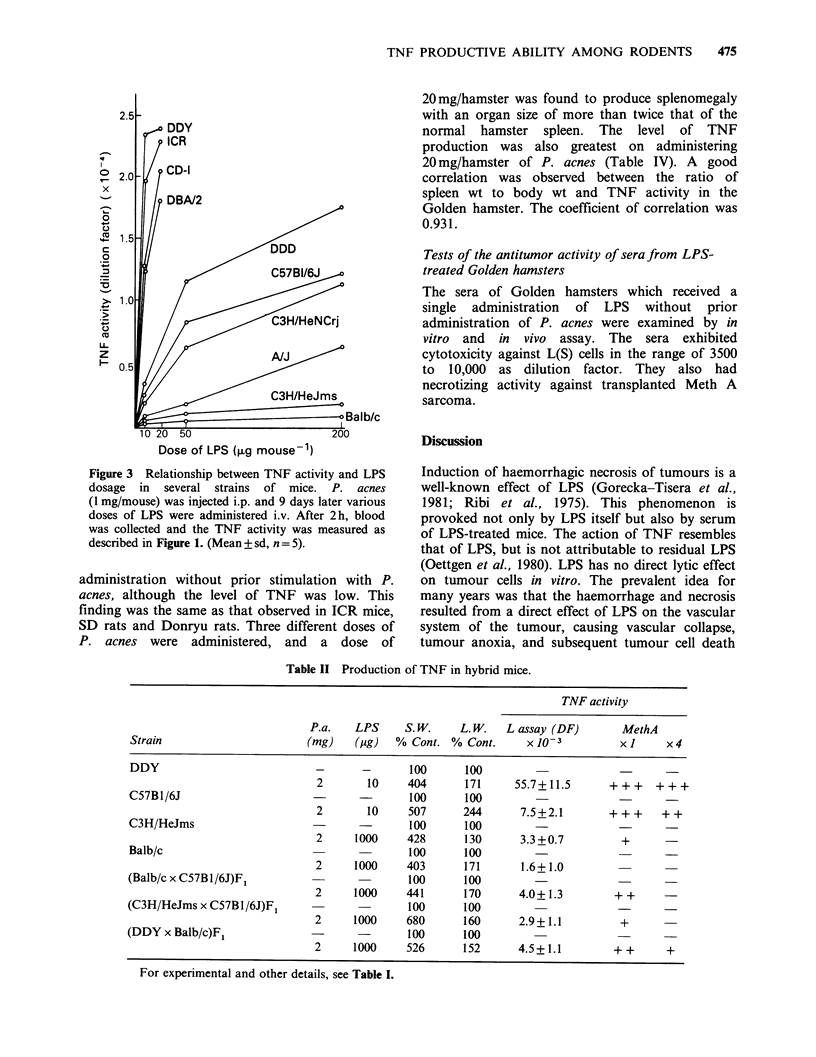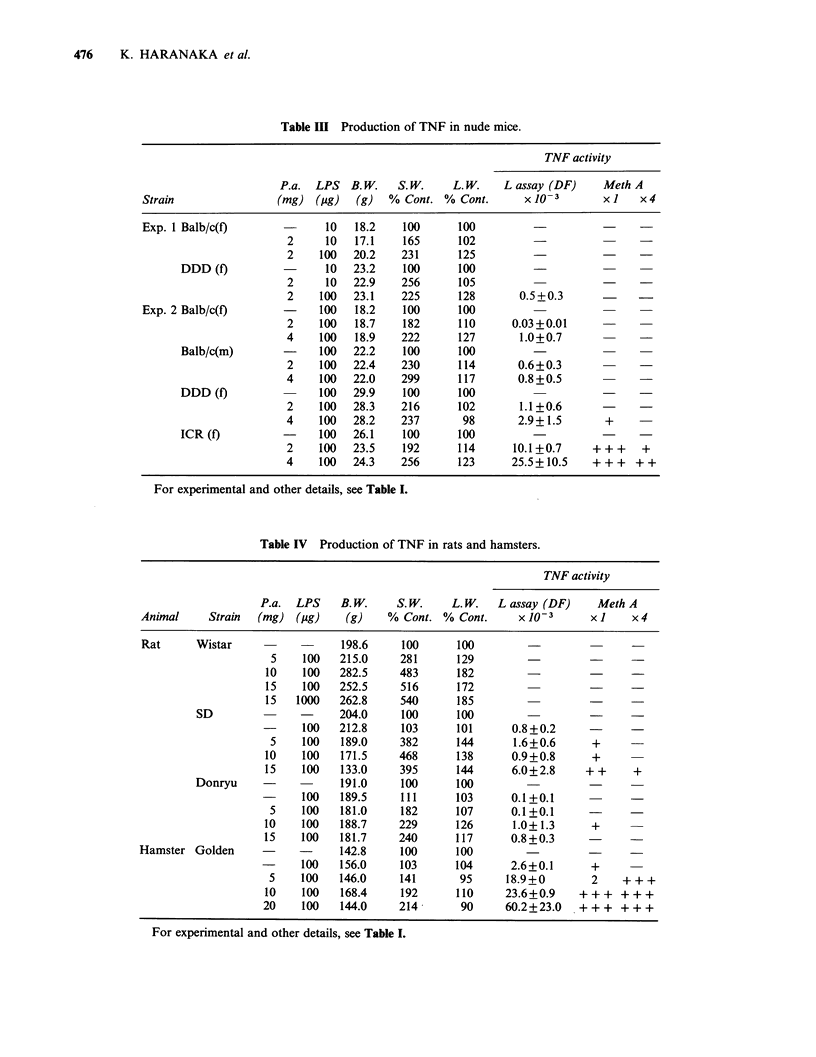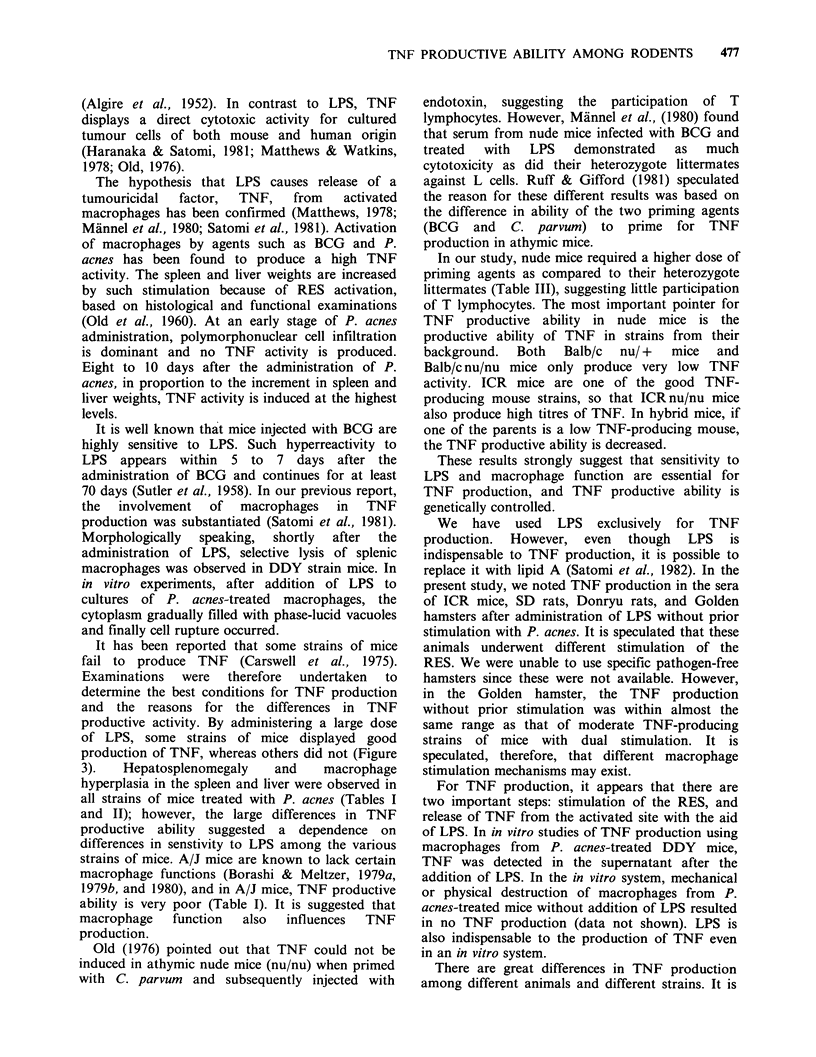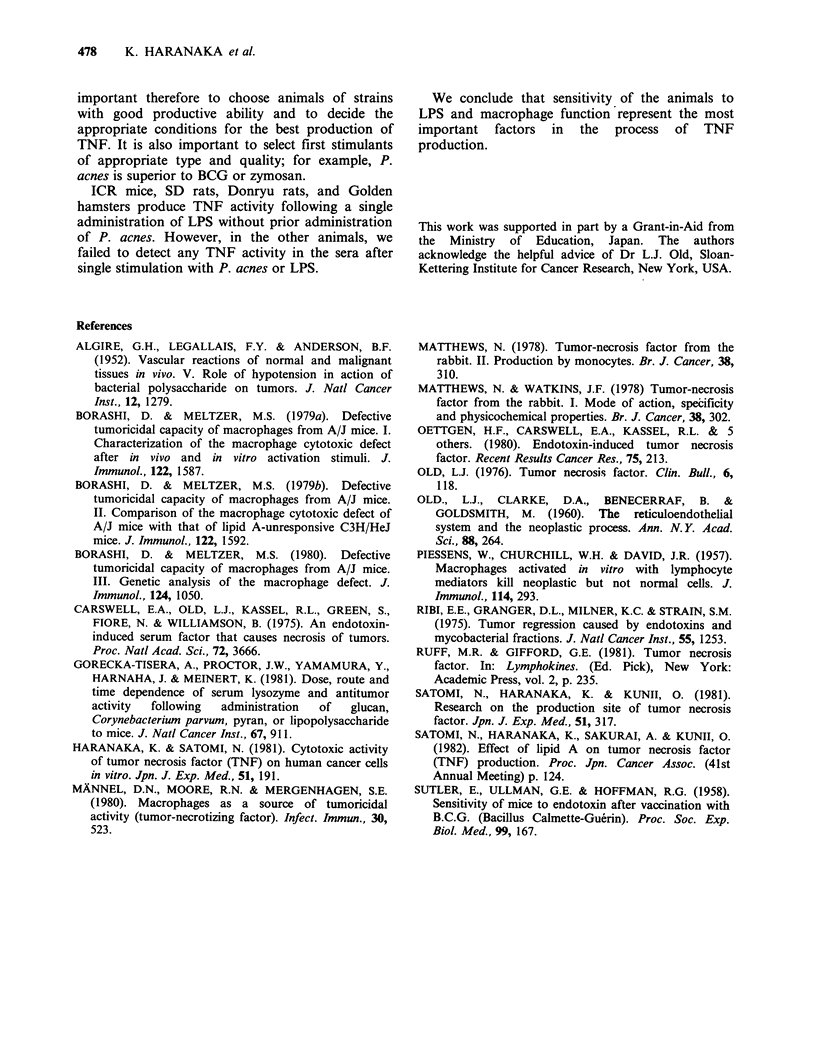# Differences in tumour necrosis factor productive ability among rodents.

**DOI:** 10.1038/bjc.1984.203

**Published:** 1984-10

**Authors:** K. Haranaka, N. Satomi, A. Sakurai

## Abstract

Large differences in tumour necrosis factor (TNF) productive ability 05337257erved among various strains of mice. DDY, CD-1, ICR and DBA/2 mice could produce a high titre of TNF activity, whereas Balb/c, C3H/HeJms and A/J mice produced a low titre of TNF activity. Administration of 200 micrograms/mouse of LPS to some strains of mice, i.e. DDD and C57B1/6J resulted in good production of TNF. ICR nu/nu mice produced the highest TNF activity among the nude mice. Balb/c nu/nu and DDD nu/nu mice exhibited very low titres of TNF activity. Nude mice required a rather higher dose of the priming agent, Propionibacterium acnes, than heterozygote littermates. Although it is commonly accepted that dual stimulation is necessary for TNF production, TNF activity was detected without the priming agent in SD rats and Golden hamsters by single injection of LPS. In these animals, much higher TNF production was observed after Propionibacterium acnes treatment than after a single injection of LPS. Large differences in TNF productive ability also existed among strains of rats. Although all animals receiving priming agents revealed hyperplasia of reticuloendothelial system, the sensitivity of the animals to LPS is considered to be the most important factor in their TNF productive ability.


*Br. J. Cancer* (1984), **50**, 471–478

### Differences in tumour necrosis factor productive ability among rodents

K. Haranaka, N. Satomi & A. Sakurai

*Department of Internal Medicine, Institute of Medical Science, University of Tokyo, Minatoku, Tokyo 108, Japan.*

**Summary** Large differences in tumour necrosis factor (TNF) productive ability were observed among various strains of mice. DDY, CD-1, ICR and DBA/2 mice could produce a high titre of TNF activity, whereas Balb/c, C3H/HeJms and A/J mice produced a low titre of TNF activity. Administration of 200 $\mu$g/mouse of LPS to some strains of mice, i.e. DDD and C57B1/6J resulted in good production of TNF. ICR nu/nu mice produced the highest TNF activity among the nude mice. Balb/c nu/nu and DDD nu/nu mice exhibited very low titres of TNF activity. Nude mice required a rather higher dose of the priming agent, *Propionibacterium acnes,* than heterozygote littermates. Although it is commonly accepted that dual stimulation is necessary for TNF production, TNF activity was detected without the priming agent in SD rats and Golden hamsters by single injection of LPS. In these animals, much higher TNF production was observed after *Propionibacterium acnes* treatment than after a single injection of LPS. Large differences in TNF productive ability also existed among strains of rats. Although all animals receiving priming agents revealed hyperplasia of reticuloendothelial system, the sensitivity of the animals to LPS is considered to be the most important factor in their TNF productive ability.

Many investigators have been fascinated by the possibility that the reticuloendothelial system (RES) may be involved in the host response to neoplastic processes. Certain agents which activate the RES, such as BCG, *Corynebacterium parvum* (*C. parvum*) and zymosan, have been found to alter the development of a number of experimental tumours (Old *et al.*, 1960; Piessens *et al.*, 1957).

Rodents can be made hypersensitive to the lethal effects of endotoxin by pretreatment with immunomodulating agents, such as B.C.G., *C. parvum* and zymosan. Serum from such hypersensitive rodents in endotoxin-induced shock has been called tumour necrosis serum (TNS). The active factor of the serum is termed tumour necrosis factor (TNF) since it causes haemorrhagic necrosis of certain transplanted solid tumours (Carswell *et al.*, 1975).

TNF exhibits a direct cytotoxicity against murine or human cancer cells *in vitro* but has no cytotoxicity against normal or embryonal cells (Haranaka & Satomi, 1981; Matthews & Watkins, 1978; Old, 1976). The *in vitro* cytotoxic activity is of particular interest because the observed properties of tumour cell sensitivity and species independence are characteristic of the *in vitro* toxic activity of activated macrophages (Oettgen *et al.*, 1980; Piessens *et al.*, 1957). Our previous studies demonstrated that TNF is produced from activated macrophages (Satomi *et al.*, 1981).

Correspondence: K. Haranaka.
Received 5 April, 1984; accepted 25 June 1984.

Carswell *et al.* (1975) reported that some strains of mice revealed a very low production of TNF. In the present study, further investigations were undertaken to elucidate the reasons why great difference in TNF productive ability exist among rodents. It is well known that dual stimulation is generally essential for the production of TNF. We examined TNF production under several conditions by varying the dose of stimulants using several strains of mice, rats, and hamsters to determine the best conditions for TNF production in rodents.

### Materials and methods

### *Animals*

The strains of mice used were as follows. DDY mice, ICR mice and ICR nu/nu mice were purchased from Shizudokyo (Shizuoka, Japan). DBA/2, DDD, Balb/c, DDD nu/nu, Balb/c nu/nu, C57B1/6J, and C3H/HeJms mice were provided by the Animal Facilities of our institute. C3H/HeNCrj and CD-1 mice were purchased from Charles River Japan (Kanagawa, Japan). Wistar rats, SD rats, Donryu rats and Golden hamsters were also purchased from Shizudokyo. The hybrid mice prepared in our laboratory were (C3H/HeJms × C57B1/6J) $F_1$, (Balb/c X C57B1/6J) $F_1$, and (DDY × Balb/c) $F_1$.

### *Vaccine*

*Propionibacterium acnes* (*P. acnes*) IID 912 was cultured in GAM broth (Nissui Pharmaceutic Co., Tokyo, Japan) under anaerobic conditions at 37°C

© The Macmillan Press Ltd., 1984

for 3 days. Bacteria were killed in 1% formalin and washed 3 times in physiological saline.

*Lipopolysaccharide (LPS)*

LPS of *Escherichia coli* 0 111:B4*w* (Difco Lab., Michigan, USA) was dissolved in physiological saline.

*Schedule of TNF production*

The time course of TNF production was studied using DDY strain mice. *P. acnes* (1, 2, or 4 mg/mouse in 0.5 ml of physiological saline) was injected i.p. as a priming agent. Nine days after the *P. acnes* injection, 10 $\mu$g/mouse of LPS was injected i.v., and 2 h after the LPS injection, blood was collected from the postorbital venous plexus with a micropipette.

In rats, different doses of *P. acnes* were administered i.p. Nine days after the administration, several doses (100 or 1000 $\mu$g/rat) of LPS were injected into the tail vein. Two hours after the LPS administration, blood was collected by heart puncture.

In the case of the hamster, *P. acnes* was also injected i.p. LPS was then administered i.v. into the postorbital venous plexus at 9 days after the *P. acnes* injection, and blood was collected by heart puncture 2 h after the LPS injection.

Serum was separated by centrifugation, and stored at $-20$°C until TNF assay *in vitro* and *in vivo*.

The body, liver and spleen wts were monitored.

As a negative control, no treatment, *P. acnes* alone or LPS alone was examined.

*Standard TNF assay* in vitro

L(S) cells, mouse fibroblast cells which are sensitive to TNF, were cultured in Eagle's MEM with heat-inactivated foetal bovine serum (10%), 100 units $ml^{-1}$ of penicillin, 100 $\mu g\, ml^{-1}$ of streptomycin, and 10 mM HEPES buffer. Serially-diluted test samples and $2 \times 10^5\, ml^{-1}$ L(S) cells were incubated in a 96 well microplate for 48 h in 5% $CO_2$ in air at 37°C. After being drawn out of the medium, the cells were fixed with methanol and stained with 0.05% methylene blue for 5 min. The dye was extracted from the cells with 3% HCl, the optical density at 665 nm was measured with a Titertek multiscan spectrophotometer (Flow Lab., Virginia, USA) and 50% cytotoxicity was assessed and expressed as the dilution factor (DF). An example of the calculation was as follows:

| | ×200 | ×400 | ×800 | ×1600 | ×3200 | ×6400 | ×12800 | (−) |
|---|---|---|---|---|---|---|---|---|
| Y [log(dilution)] | 2.301 | 2.602 | 2.903 | 3.204 | 3.505 | 3.806 | 4.107 | |
| X ($OD_{665}$) | 0.089 | 0.126 | 0.249 | 0.437 | 0.575 | 0.637 | 0.650 | 0.663 |

$Y = 2.377 + 1.949X$, $R = 0.997$ (between ×400 and ×3200).

50% $OD_{665} = 0.332$, $Y = 2.377 + 1.949 \times 0.332 = 3.024$.

From this formula, the dilution factor was determined as $10^{3.024} = 1057$. L(R) cells, mouse fibroblast cells insensitive to TNF, were also examined as a negative control to exclude other bioactive substances.

*Standard TNF assay* in vivo

Five million Meth A sarcoma cells were injected i.d. into the flank of Balb/c mice. Seven days after the transplantation, 0.5 ml of test sample was injected i.v. and the degree of tumour necrosis was assessed 24 h later as follows: grade (−), no changes; grade (+), slight necrosis; grade (++), moderate necrosis (central necrosis extending over ~50% of the tumour surface); and grade (+++), extensive necrosis (massive necrosis leaving at most only a small rim of viable tumour tissue).

### Results

*Time course of TNF production, spleen weight, and liver weight after administration of* P. acnes *and LPS*

Between 8 and 10 days after *P. acnes* administration, the highest level of TNF production was observed in DDY strain mice (Figure 1). Hepatosplenomegaly was most marked on the 12th day, and continued for a long time.

*Time course of TNF production after administration of LPS*

The production of TNF was greatest between 90 and 120 min after LPS administration in *P. acnes*-primed mice (Figure 2). During this period, some of the mice suffered endotoxin shock and died. Two hours after the administration of LPS, the best TNF production was observed in DDY strain mice.

*Production of TNF in several strains of mice*

The TNF production and weights of the spleen and liver in several strains of mice are shown in Table I. DDY, CD-1, ICR and DBA/2 strain mice exhibited the highest TNF production among the mice tested. A/J, C3H/HeJms and Balb/c strain mice showed a low TNF production. In DDY, CD-1, ICR, DBA/2, DDD, C57Bl/6J and C3H/HeNCrj mice, hepatosplenomegaly was observed to almost the same degree, but in A/J, C3H/HeJms and Balb/c

**Figure 1** Time course of TNF production, liver and spleen weights after *Propionibacterium acnes* administration. *P. acnes* was injected i.p. and 10 μg LPS/mouse was administered at each point. Two hours later, blood was collected from the postorbital venous plexus. The liver (×) and spleen (□) weights were measured after blood collection. TNF activity was assessed from the dilution factor which revealed 50% cytotoxicity against L(S) cells (○). ($n = 5$).

**Figure 2** Time course of TNF production after LPS administration. *Propionibacterium acnes* (1 mg/mouse) had been injected i.p. into DDY mice 9 days previously. Blood was then collected at several times after LPS (10 μg/mouse) administration. TNF activity was assayed as described in **Figure 1.** (Mean ± sd, $n = 5$).

mice, the hepatosplenomegaly was not so marked in comparison with the other strains. Administration of a large dose of *P. acnes* to these latter 3 strains brought about hepatosplenomegaly, but the TNF production did not increase so much.

In the high TNF-producing group (DDY, CD-1, ICR and DBA/2 mice), a small dose of LPS induced a high level of TNF production. In the moderate TNF-producing group (DDD, C57Bl/6J and C3H/HeNCrj mice), a slightly larger dose of LPS than in the high TNF-producing group was required to achieve a good TNF production. In the low TNF-producing group (A/J, C3H/HeJms and Balb/c mice), even when 2 mg/mouse of *P. acnes* and 200–1000 μg/mouse of LPS were administered, the mice revealed only slight TNF production (Figure 3). In these experiments with mice, we were unable to detect TNF production by single stimulation with LPS except in the ICR mice.

With hybrid mice, the TNF production was affected by the parent mouse strains. Hybrids with Balb/c or C3H/HeJms produced low levels of TNF activity even if they were crossbred with high TNF-producing mice (Table II).

TNF production and weights of the spleen and liver of nude mice are shown in Table III. In the nude mice, the hepatosplenomegaly was not so marked compared with other strains of mice at 1 mg/mouse of *P. acnes* administration. Therefore, 2 or 4 mg/mouse of *P. acnes* was injected. In Balb/c nu/nu mice, no TNF activity could be detected at a small dose of *P. acnes*. In both Balb/c and DDD nu/nu mice, TNF activity was detected in the sera but its activity was low on administration of 4 mg/mouse of *P. acnes*. In ICR nu/nu mice, TNF activity could be detected to almost the same degree as in ICR nu/+ mice following 2–4 mg/mouse of *P. acnes* administration.

### *TNF production in rats*

The results for TNF production in rats are summarized in Table IV. Donryu rats, Wistar rats, and SD rats revealed almost the same degree of increment in spleen and liver weights 9 days after administration of *P. acnes*. Two hours after LPS injection, SD and Donryu rats showed endotoxin-shock, but Wistar rats did not suffer endotoxin

**Table I** Production of TNF in several strains of mice.

| Strain | P.a.[a] (mg) | LPS[b] (μg) | S.W.[c] % Cont. | L.W.[d] % Cont. | TNF activity L assay (DF)[e] × 10⁻³ | MethA[f] ×1 | ×4 |
|---|---|---|---|---|---|---|---|
| DDY | — | — | 100 | 100 | — | — | — |
| | — | 10 | 246 | 113 | — | — | — |
| | 1 | 10 | 264 | 130 | 18.2±10.9 | +++ | ++ |
| | 2 | 10 | 404 | 171 | 55.7±11.6 | +++ | +++ |
| CD-1 | — | — | 100 | 100 | — | — | — |
| | — | 10 | 102 | 107 | — | — | — |
| | 1 | 10 | 432 | 144 | 13.6±6.5 | +++ | ++ |
| | 2 | 10 | 825 | 261 | 46.1±9.7 | +++ | +++ |
| ICR | — | — | 100 | 100 | — | — | — |
| | — | 10 | 116 | 109 | 1.2±0.5 | + | — |
| | 1 | 10 | 311 | 202 | 20.0±17.5 | +++ | ++ |
| | 2 | 10 | 361 | 206 | 46.4±7.9 | +++ | +++ |
| DBA/2 | — | — | 100 | 100 | — | — | — |
| | — | 10 | 96 | 114 | — | — | — |
| | 1 | 10 | 218 | 157 | 12.3±7.1 | +++ | ++ |
| | 2 | 10 | 395 | 254 | 21.8±3.5 | +++ | ++ |
| DDD | — | — | 100 | 100 | — | — | — |
| | — | 10 | 99 | 96 | — | — | — |
| | 1 | 10 | 201 | 153 | 3.5±3.3 | + | — |
| | 2 | 10 | 322 | 199 | 12.5±0.6 | +++ | + |
| C57B1/6J | — | — | 100 | 100 | — | — | — |
| | — | 10 | 122 | 112 | — | — | — |
| | 1 | 10 | 338 | 177 | 2.8±2.0 | + | — |
| | 2 | 10 | 507 | 244 | 7.5±2.1 | ++ | + |
| C3H/HeNCrj | — | — | 100 | 100 | — | — | — |
| | — | 10 | 93 | 101 | — | — | — |
| | 1 | 10 | 264 | 120 | 1.2±0.5 | + | — |
| | 2 | 10 | 427 | 130 | 3.3±0.7 | ++ | — |
| C3H/HeJms | — | — | 100 | 100 | — | — | — |
| | — | 10 | 121 | 110 | — | — | — |
| | 1 | 10 | 220 | 126 | 0.6±0.4 | — | — |
| | 2 | 10 | 391 | 185 | 2.3±1.5 | + | — |
| A/J | — | — | 100 | 100 | — | — | — |
| | — | 10 | 119 | 115 | — | — | — |
| | 1 | 10 | 192 | 150 | 0.05±0.03 | — | — |
| | 2 | 10 | 424 | 216 | 3.7±2.0 | + | — |
| Balb/c | — | — | 100 | 100 | — | — | — |
| | — | 10 | 103 | 97 | — | — | — |
| | 1 | 10 | 241 | 138 | 0.09±0.07 | — | — |
| | 2 | 10 | 451 | 228 | 3.0±1.9 | + | — |

*P. acnes*[a] (0, 1 or 2 mg/mouse) was injected i.p. 9 days prior to LPS[b] (0 or 10 μg/mouse) administration. The organ weights, S.W.[c] (spleen wt) and L.W.[d] (liver wt), and TNF activity were assayed as described in **Figure 1.** The TNF activity was expressed as the dilution factor (DF) by L assay[e], and Meth A assay[f]. Seven days after $5 \times 10^5$ of Meth A transplantation, 0.5 ml of sample or 4 times diluted sample was injected i.v. After 24 h, the degree of tumor necrosis was graded. (DF: mean±sd, $n=5$, assayed individually).

shock even when 2000 μg of LPS was injected. TNF activity was detected in the sera of SD and Donryu rats, but not in Wistar rats even when a large dose of LPS was administered.

In SD or Donryu rats, TNF activity was detected in the sera after a single injection of LPS without *P. acnes* stimulation.

*TNF production in hamsters*

In Golden hamsters, the spleen and liver weights did not increase markedly after administration of *P. acnes* when compared to mice or rats, but TNF production was greatest among the animals examined. TNF was produced after LPS

**Figure 3** Relationship between TNF activity and LPS dosage in several strains of mice. *P. acnes* (1 mg/mouse) was injected i.p. and 9 days later various doses of LPS were administered i.v. After 2 h, blood was collected and the TNF activity was measured as described in **Figure 1.** (Mean ± sd, $n = 5$).

administration without prior stimulation with *P. acnes,* although the level of TNF was low. This finding was the same as that observed in ICR mice, SD rats and Donryu rats. Three different doses of *P. acnes* were administered, and a dose of 20 mg/hamster was found to produce splenomegaly with an organ size of more than twice that of the normal hamster spleen. The level of TNF production was also greatest on administering 20 mg/hamster of *P. acnes* (Table IV). A good correlation was observed between the ratio of spleen wt to body wt and TNF activity in the Golden hamster. The coefficient of correlation was 0.931.

### *Tests of the antitumor activity of sera from LPS-treated Golden hamsters*

The sera of Golden hamsters which received a single administration of LPS without prior administration of *P. acnes* were examined by *in vitro* and *in vivo* assay. The sera exhibited cytotoxicity against L(S) cells in the range of 3500 to 10,000 as dilution factor. They also had necrotizing activity against transplanted Meth A sarcoma.

### Discussion

Induction of haemorrhagic necrosis of tumours is a well-known effect of LPS (Gorecka-Tisera *et al.*, 1981; Ribi *et al.*, 1975). This phenomenon is provoked not only by LPS itself but also by serum of LPS-treated mice. The action of TNF resembles that of LPS, but is not attributable to residual LPS (Oettgen *et al.*, 1980). LPS has no direct lytic effect on tumour cells *in vitro.* The prevalent idea for many years was that the haemorrhage and necrosis resulted from a direct effect of LPS on the vascular system of the tumour, causing vascular collapse, tumour anoxia, and subsequent tumour cell death

**Table II** Production of TNF in hybrid mice.

| | | | | | TNF activity | | |
|---|---|---|---|---|---|---|---|
| *Strain* | *P.a. (mg)* | *LPS (μg)* | *S.W. % Cont.* | *L.W. % Cont.* | *L assay (DF) ×10⁻³* | *MethA ×1* | *×4* |
| DDY | – | – | 100 | 100 | — | — | — |
| | 2 | 10 | 404 | 171 | 55.7±11.5 | +++ | +++ |
| C57Bl/6J | — | — | 100 | 100 | — | — | — |
| | 2 | 10 | 507 | 244 | 7.5±2.1 | +++ | ++ |
| C3H/HeJms | — | — | 100 | 100 | — | — | — |
| | 2 | 1000 | 428 | 130 | 3.3±0.7 | + | — |
| Balb/c | — | — | 100 | 100 | — | — | — |
| | 2 | 1000 | 403 | 171 | 1.6±1.0 | — | — |
| (Balb/c × C57Bl/6J)$F_1$ | — | — | 100 | 100 | — | — | — |
| | 2 | 1000 | 441 | 170 | 4.0±1.3 | ++ | — |
| (C3H/HeJms × C57Bl/6J)$F_1$ | — | — | 100 | 100 | — | — | — |
| | 2 | 1000 | 680 | 160 | 2.9±1.1 | + | — |
| (DDY × Balb/c)$F_1$ | — | — | 100 | 100 | — | — | — |
| | 2 | 1000 | 526 | 152 | 4.5±1.1 | ++ | + |

For experimental and other details, see **Table I.**

**Table III** Production of TNF in nude mice.

| Strain | P.a. (mg) | LPS (μg) | B.W. (g) | S.W. % Cont. | L.W. % Cont. | TNF activity L assay (DF) $\times 10^{-3}$ | TNF activity Meth A ×1 | TNF activity Meth A ×4 |
|---|---|---|---|---|---|---|---|---|
| Exp. 1 Balb/c(f) | — | 10 | 18.2 | 100 | 100 | — | — | — |
| | 2 | 10 | 17.1 | 165 | 102 | — | — | — |
| | 2 | 100 | 20.2 | 231 | 125 | — | — | — |
| DDD (f) | — | 10 | 23.2 | 100 | 100 | — | — | — |
| | 2 | 10 | 22.9 | 256 | 105 | — | — | — |
| | 2 | 100 | 23.1 | 225 | 128 | 0.5±0.3 | — | — |
| Exp. 2 Balb/c(f) | — | 100 | 18.2 | 100 | 100 | — | — | — |
| | 2 | 100 | 18.7 | 182 | 110 | 0.03±0.01 | — | — |
| | 4 | 100 | 18.9 | 222 | 127 | 1.0±0.7 | — | — |
| Balb/c(m) | — | 100 | 22.2 | 100 | 100 | — | — | — |
| | 2 | 100 | 22.4 | 230 | 114 | 0.6±0.3 | — | — |
| | 4 | 100 | 22.0 | 299 | 117 | 0.8±0.5 | — | — |
| DDD (f) | — | 100 | 29.9 | 100 | 100 | — | — | — |
| | 2 | 100 | 28.3 | 216 | 102 | 1.1±0.6 | — | — |
| | 4 | 100 | 28.2 | 237 | 98 | 2.9±1.5 | + | — |
| ICR (f) | — | 100 | 26.1 | 100 | 100 | — | — | — |
| | 2 | 100 | 23.5 | 192 | 114 | 10.1±0.7 | +++ | + |
| | 4 | 100 | 24.3 | 256 | 123 | 25.5±10.5 | +++ | ++ |

For experimental and other details, see **Table I.**

**Table IV** Production of TNF in rats and hamsters.

| Animal | Strain | P.a. (mg) | LPS (μg) | B.W. (g) | S.W. % Cont. | L.W. % Cont. | TNF activity L assay (DF) $\times 10^{-3}$ | TNF activity Meth A ×1 | TNF activity Meth A ×4 |
|---|---|---|---|---|---|---|---|---|---|
| Rat | Wistar | — | — | 198.6 | 100 | 100 | — | — | — |
| | | 5 | 100 | 215.0 | 281 | 129 | — | — | — |
| | | 10 | 100 | 282.5 | 483 | 182 | — | — | — |
| | | 15 | 100 | 252.5 | 516 | 172 | — | — | — |
| | | 15 | 1000 | 262.8 | 540 | 185 | — | — | — |
| | SD | — | — | 204.0 | 100 | 100 | — | — | — |
| | | — | 100 | 212.8 | 103 | 101 | 0.8±0.2 | — | — |
| | | 5 | 100 | 189.0 | 382 | 144 | 1.6±0.6 | + | — |
| | | 10 | 100 | 171.5 | 468 | 138 | 0.9±0.8 | + | — |
| | | 15 | 100 | 133.0 | 395 | 144 | 6.0±2.8 | ++ | + |
| | Donryu | — | — | 191.0 | 100 | 100 | — | — | — |
| | | — | 100 | 189.5 | 111 | 103 | 0.1±0.1 | — | — |
| | | 5 | 100 | 181.0 | 182 | 107 | 0.1±0.1 | — | — |
| | | 10 | 100 | 188.7 | 229 | 126 | 1.0±1.3 | + | — |
| | | 15 | 100 | 181.7 | 240 | 117 | 0.8±0.3 | — | — |
| Hamster | Golden | — | — | 142.8 | 100 | 100 | — | — | — |
| | | — | 100 | 156.0 | 103 | 104 | 2.6±0.1 | + | — |
| | | 5 | 100 | 146.0 | 141 | 95 | 18.9±0 | 2 | +++ |
| | | 10 | 100 | 168.4 | 192 | 110 | 23.6±0.9 | +++ | +++ |
| | | 20 | 100 | 144.0 | 214 | 90 | 60.2±23.0 | +++ | +++ |

For experimental and other details, see **Table I.**

(Algire *et al.*, 1952). In contrast to LPS, TNF displays a direct cytotoxic activity for cultured tumour cells of both mouse and human origin (Haranaka & Satomi, 1981; Matthews & Watkins, 1978; Old, 1976).

The hypothesis that LPS causes release of a tumouricidal factor, TNF, from activated macrophages has been confirmed (Matthews, 1978; Männel *et al.*, 1980; Satomi *et al.*, 1981). Activation of macrophages by agents such as BCG and *P. acnes* has been found to produce a high TNF activity. The spleen and liver weights are increased by such stimulation because of RES activation, based on histological and functional examinations (Old *et al.*, 1960). At an early stage of *P. acnes* administration, polymorphonuclear cell infiltration is dominant and no TNF activity is produced. Eight to 10 days after the administration of *P. acnes,* in proportion to the increment in spleen and liver weights, TNF activity is induced at the highest levels.

It is well known that mice injected with BCG are highly sensitive to LPS. Such hyperreactivity to LPS appears within 5 to 7 days after the administration of BCG and continues for at least 70 days (Sutler *et al.*, 1958). In our previous report, the involvement of macrophages in TNF production was substantiated (Satomi *et al.*, 1981). Morphologically speaking, shortly after the administration of LPS, selective lysis of splenic macrophages was observed in DDY strain mice. In *in vitro* experiments, after addition of LPS to cultures of *P. acnes*-treated macrophages, the cytoplasm gradually filled with phase-lucid vacuoles and finally cell rupture occurred.

It has been reported that some strains of mice fail to produce TNF (Carswell *et al.*, 1975). Examinations were therefore undertaken to determine the best conditions for TNF production and the reasons for the differences in TNF productive activity. By administering a large dose of LPS, some strains of mice displayed good production of TNF, whereas others did not (Figure 3). Hepatosplenomegaly and macrophage hyperplasia in the spleen and liver were observed in all strains of mice treated with *P. acnes* (Tables I and II); however, the large differences in TNF productive ability suggested a dependence on differences in senstivity to LPS among the various strains of mice. A/J mice are known to lack certain macrophage functions (Borashi & Meltzer, 1979*a*, 1979*b*, and 1980), and in A/J mice, TNF productive ability is very poor (Table I). It is suggested that macrophage function also influences TNF production.

Old (1976) pointed out that TNF could not be induced in athymic nude mice (nu/nu) when primed with *C. parvum* and subsequently injected with endotoxin, suggesting the participation of T lymphocytes. However, Männel *et al.*, (1980) found that serum from nude mice infected with BCG and treated with LPS demonstrated as much cytotoxicity as did their heterozygote littermates against L cells. Ruff & Gifford (1981) speculated the reason for these different results was based on the difference in ability of the two priming agents (BCG and *C. parvum*) to prime for TNF production in athymic mice.

In our study, nude mice required a higher dose of priming agents as compared to their heterozygote littermates (Table III), suggesting little participation of T lymphocytes. The most important pointer for TNF productive ability in nude mice is the productive ability of TNF in strains from their background. Both Balb/c nu/+ mice and Balb/c nu/nu mice only produce very low TNF activity. ICR mice are one of the good TNF-producing mouse strains, so that ICR nu/nu mice also produce high titres of TNF. In hybrid mice, if one of the parents is a low TNF-producing mouse, the TNF productive ability is decreased.

These results strongly suggest that sensitivity to LPS and macrophage function are essential for TNF production, and TNF productive ability is genetically controlled.

We have used LPS exclusively for TNF production. However, even though LPS is indispensable to TNF production, it is possible to replace it with lipid A (Satomi *et al.*, 1982). In the present study, we noted TNF production in the sera of ICR mice, SD rats, Donryu rats, and Golden hamsters after administration of LPS without prior stimulation with *P. acnes*. It is speculated that these animals underwent different stimulation of the RES. We were unable to use specific pathogen-free hamsters since these were not available. However, in the Golden hamster, the TNF production without prior stimulation was within almost the same range as that of moderate TNF-producing strains of mice with dual stimulation. It is speculated, therefore, that different macrophage stimulation mechanisms may exist.

For TNF production, it appears that there are two important steps: stimulation of the RES, and release of TNF from the activated site with the aid of LPS. In *in vitro* studies of TNF production using macrophages from *P. acnes*-treated DDY mice, TNF was detected in the supernatant after the addition of LPS. In the *in vitro* system, mechanical or physical destruction of macrophages from *P. acnes*-treated mice without addition of LPS resulted in no TNF production (data not shown). LPS is also indispensable to the production of TNF even in an *in vitro* system.

There are great differences in TNF production among different animals and different strains. It is important therefore to choose animals of strains with good productive ability and to decide the appropriate conditions for the best production of TNF. It is also important to select first stimulants of appropriate type and quality; for example, *P. acnes* is superior to BCG or zymosan.

ICR mice, SD rats, Donryu rats, and Golden hamsters produce TNF activity following a single administration of LPS without prior administration of *P. acnes*. However, in the other animals, we failed to detect any TNF activity in the sera after single stimulation with *P. acnes* or LPS.

We conclude that sensitivity of the animals to LPS and macrophage function represent the most important factors in the process of TNF production.

This work was supported in part by a Grant-in-Aid from the Ministry of Education, Japan. The authors acknowledge the helpful advice of Dr L.J. Old, Sloan-Kettering Institute for Cancer Research, New York, USA.

### References

ALGIRE, G.H., LEGALLAIS, F.Y. & ANDERSON, B.F. (1952). Vascular reactions of normal and malignant tissues *in vivo*. V. Role of hypotension in action of bacterial polysaccharide on tumors. *J. Natl Cancer Inst.*, **12,** 1279.

BORASHI, D. & MELTZER, M.S. (1979*a*). Defective tumoricidal capacity of macrophages from A/J mice. I. Characterization of the macrophage cytotoxic defect after *in vivo* and *in vitro* activation stimuli. *J. Immunol.*, **122,** 1587.

BORASHI, D. & MELTZER, M.S. (1979*b*). Defective tumoricidal capacity of macrophages from A/J mice. II. Comparison of the macrophage cytotoxic defect of A/J mice with that of lipid A-unresponsive C3H/HeJ mice. *J. Immunol.*, **122,** 1592.

BORASHI, D. & MELTZER, M.S. (1980). Defective tumoricidal capacity of macrophages from A/J mice. III. Genetic analysis of the macrophage defect. *J. Immunol.*, **124,** 1050.

CARSWELL, E.A., OLD, L.J., KASSEL, R.L., GREEN, S., FIORE, N. & WILLIAMSON, B. (1975). An endotoxin-induced serum factor that causes necrosis of tumors. *Proc. Natl Acad. Sci.*, **72,** 3666.

GORECKA-TISERA, A., PROCTOR, J.W., YAMAMURA, Y., HARNAHA, J. & MEINERT, K. (1981). Dose, route and time dependence of serum lysozyme and antitumor activity following administration of glucan, *Corynebacterium parvum*, pyran, or lipopolysaccharide to mice. *J. Natl Cancer Inst.*, **67,** 911.

HARANAKA, K. & SATOMI, N. (1981). Cytotoxic activity of tumor necrosis factor (TNF) on human cancer cells *in vitro*. *Jpn. J. Exp. Med.*, **51,** 191.

MÄNNEL, D.N., MOORE, R.N. & MERGENHAGEN, S.E. (1980). Macrophages as a source of tumoricidal activity (tumor-necrotizing factor). *Infect. Immun.*, **30,** 523.

MATTHEWS, N. (1978). Tumor-necrosis factor from the rabbit. II. Production by monocytes. *Br. J. Cancer*, **38,** 310.

MATTHEWS, N. & WATKINS, J.F. (1978) Tumor-necrosis factor from the rabbit. I. Mode of action, specificity and physicochemical properties. *Br. J. Cancer*, **38,** 302.

OETTGEN, H.F., CARSWELL, E.A., KASSEL, R.L. & 5 others. (1980). Endotoxin-induced tumor necrosis factor. *Recent Results Cancer Res.*, **75,** 213.

OLD, L.J. (1976). Tumor necrosis factor. *Clin. Bull.*, **6,** 118.

OLD., L.J., CLARKE, D.A., BENECERRAF, B. & GOLDSMITH, M. (1960). The reticuloendothelial system and the neoplastic process. *Ann. N.Y. Acad. Sci.*, **88,** 264.

PIESSENS, W., CHURCHILL, W.H. & DAVID, J.R. (1957). Macrophages activated *in vitro* with lymphocyte mediators kill neoplastic but not normal cells. *J. Immunol.*, **114,** 293.

RIBI, E.E., GRANGER, D.L., MILNER, K.C. & STRAIN, S.M. (1975). Tumor regression caused by endotoxins and mycobacterial fractions. *J. Natl Cancer Inst.*, **55,** 1253.

RUFF, M.R. & GIFFORD, G.E. (1981). Tumor necrosis factor. In: *Lymphokines*. (Ed. Pick), New York: Academic Press, vol. 2, p. 235.

SATOMI, N., HARANAKA, K. & KUNII, O. (1981). Research on the production site of tumor necrosis factor. *Jpn. J. Exp. Med.*, **51,** 317.

SATOMI, N., HARANAKA, K., SAKURAI, A. & KUNII, O. (1982). Effect of lipid A on tumor necrosis factor (TNF) production. *Proc. Jpn. Cancer Assoc.* (41st Annual Meeting) p. 124.

SUTLER, E., ULLMAN, G.E. & HOFFMAN, R.G. (1958). Sensitivity of mice to endotoxin after vaccination with B.C.G. (Bacillus Calmette-Guérin). *Proc. Soc. Exp. Biol. Med.*, **99,** 167.